# Biomarkers, clinical status, and cognitive performance in a Caribbean Hispanic cohort

**DOI:** 10.1002/alz.093556

**Published:** 2025-01-09

**Authors:** Lawrence S. Honig, Min Suk Kang, Dolly Reyes‐Dumeyer, Yahaira Franco, Martin Medrano, Angel Piriz, Patricia Recio, Diones Rivera Mejia, Belisa Soriano, Rafael A. Lantigua, Richard Mayeux

**Affiliations:** ^1^ Columbia University Irving Medical Center, New York, NY USA; ^2^ Clinica Corominas, Santiago Dominican Republic; ^3^ School of Medicine, Pontificia Universidad Catolica Madre y Maestra, Santiago Dominican Republic; ^4^ CEDIMAT, Santo Domingo Dominican Republic; ^5^ Universidad Pedro Henríquez Urena, Santo Domingo Dominican Republic

## Abstract

**Background:**

Plasma biomarkers may be utilized to assist in diagnosis of Alzheimer's disease. However, in a cohort of Caribbean Hispanic individuals we have shown that there are both individuals who are biomarker positive but without dementia, and biomarker negative but diagnosed with dementia. Here we examine education and neuropsychological testing performance in these subgroups with biomarker‐inconsistent dementia diagnosis.

**Method:**

Adults of Caribbean Hispanic ethnicity were recruited in both New York City and the Dominican Republic. The group reported here includes 1173 individuals. Plasma biomarkers including AB40, AB42, total tau, phosphorylated‐tau181, Neurofilament light chain (NfL), and Glial Fibrillary Acidic Protein (GFAP) were measured in samples processed, stored at ‐80°C, and thawed for analysis. Measurements were made using the Simoa Quanterix HD‐X platform.

**Result:**

In this group of 1172 individuals, 885 (75.5%) were assessed as clinically without dementia, and 288 (24.5%) as clinically with dementia. Of those without dementia, 627 (70.8%) were biomarker negative, and 258 (29.2%) were biomarker positive for p‐tau181. Of those with dementia, 173 (60.1%) were biomarker positive, and 115 (39.9%) were biomarker negative for p‐tau181. For those without dementia, there was no significant difference in education level or performance on most neuropsychological measures between those biomarker positive or negative, except that delayed recall on the SRT word‐list‐learning test was lower in those with higher p‐tau181 (mean 4.25 ± 2.27 vs. 4.65 ± 1.98; p=0.009). For those with dementia, education was significantly lower in those that were p‐tau181 negative (2.87 ± 3.36 vs. 4.18 ± 4.73 yr;p=0.011), but these individuals also performed significantly better in neuropsychological tests including SRT immediate (p=0.003) and delayed recall (p<0.001), orientation (p=0.002), and Benton‐matching (p=0.012).

**Conclusion:**

Plasma biomarkers may perform well in diagnosis of dementia in clinic populations, but less well in underserved populations, with more persons without dementia but with positive biomarkers, and with dementia but with negative biomarkers. For those without dementia but with positive biomarkers, likely many have pre‐symptomatic Alzheimer's; lower performance on memory tests supports this explanation. For those with dementia, but without positive biomarkers, explanations may include lower cognitive reserve/educational attainment level, increased comorbidities affecting functional status, and the presence of non‐Alzheimer's brain disorders.

